# Age-Related Changes in Dynamic Postural Control and Attentional Demands are Minimally Affected by Local Muscle Fatigue

**DOI:** 10.3389/fnagi.2015.00257

**Published:** 2016-01-21

**Authors:** Anthony Remaud, Cécile Thuong-Cong, Martin Bilodeau

**Affiliations:** ^1^Aging and Movement Laboratory, Bruyère Research InstituteOttawa, ON, Canada; ^2^Faculty of Health Sciences, School of Rehabilitation Sciences, University of OttawaOttawa, ON, Canada; ^3^Faculty of Health Sciences, School of Human Kinetics, University of OttawaOttawa, ON, Canada

**Keywords:** aging, muscle fatigue, dynamic postural control, dual-task, cross-correlation analysis, posturography

## Abstract

Normal aging results in alterations in the visual, vestibular and somtaosensory systems, which in turn modify the control of balance. Muscle fatigue may exacerbate these age-related changes in sensory and motor functions, and also increase the attentional demands associated with dynamic postural control. The purpose of this study was to investigate the effect of aging on dynamic postural control and posture-related attentional demands before and after a plantar flexor fatigue protocol. Participants (young adults: *n* = 15; healthy seniors: *n* = 13) performed a dynamic postural task along the antero-posterior (AP) and the medio-lateral (ML) axes, with and without the addition of a simple reaction time (RT) task. The dynamic postural task consisted in following a moving circle on a computer screen with the representation of the center of pressure (COP). This protocol was repeated before and after a fatigue task where ankle plantar flexor muscles were targeted. The mean COP-target distance and the mean COP velocity were calculated for each trial. Cross-correlation analyses between the COP and target displacements were also performed. RTs were recorded during dual-task trials. Results showed that while young adults adopted an anticipatory control mode to move their COP as close as possible to the target center, seniors adopted a reactive control mode, lagging behind the target center. This resulted in longer COP-target distance and higher COP velocity in the latter group. Concurrently, RT increased more in seniors when switching from static stance to dynamic postural conditions, suggesting potential alterations in the central nervous system (CNS) functions. Finally, plantar flexor muscle fatigue and dual-tasking had only minor effects on dynamic postural control of both young adults and seniors. Future studies should investigate why the fatigue-induced changes in quiet standing postural control do not seem to transfer to dynamic balance tasks.

## Introduction

The accomplishment of daily living activities, such as leaning over to grab an object, requires adequate postural control to maintain the center of gravity within the base of support (BOS). For this purpose, the central nervous system (CNS) must continuously integrate different sources of sensory information (mainly from the visual, vestibular and somatosensory systems), while generating appropriate motor commands to perform balance corrections (Diener and Dichgans, 1988). During dynamic conditions, postural balance is threatened by internal and/or external perturbations. These perturbations can occur in a predictable or unpredictable manner, thus triggering anticipatory (Bouisset and Zattara, 1981; Massion, 1992) or compensatory (Nashner and Cordo, 1981) postural responses.

Normal aging results in alterations in the above-mentioned sensory systems (Sturnieks et al., 2008), which in turn modify the control of balance and may lead to falls and severe injuries (Tinetti et al., 1988). For example, seniors can experience visual impairment, especially a loss in contrast sensitivity and depth perception, which increase postural sway and the risk of falls (Lord, 2006). Aging is also associated with an overall decline in semicircular canals and otolith function that alters vestibular functions (Agrawal et al., 2012). In addition, proprioception tends to deteriorate with age, with studies showing a decline in joint position and motion sense in the elderly (Goble et al., 2009), likely due to a loss of large myelinated sensory fibers and receptors in the periphery (Shaffer and Harrison, 2007). In parallel, aging is also accompanied by multiple changes in neuromuscular structures and function, particularly a loss of spinal motoneurons and a reduction in muscle fiber number and size, which impair motor performance (Aagaard et al., 2010) and decrease the ability of seniors to adapt to postural perturbations and recover from a loss of balance (Bugnariu and Sveistrup, 2006).

Certain factors may exacerbate age-related changes in sensory and motor functions. For example, sustained muscle activity leads to muscle fatigue, i.e., a transient decrease in the capacity to perform physical actions (Enoka and Duchateau, 2008). Muscle fatigue is a complex phenomenon which implicates a variety of physiological processes occurring at different levels, from the motor cortex to muscle contractile proteins (Gandevia, 2001; Hunter et al., 2004; Boyas and Guével, 2011). Numerous studies have reported postural control impairments after general and local fatiguing muscle activity in young adults (for a review, see Paillard, 2012). Since plantar flexor muscles play a major role in maintaining balance (Loram et al., 2005), many researchers have focused their work on the effects of plantar flexor fatiguing exercise on postural control (e.g., Yaggie and McGregor, 2002; Harkins et al., 2005; Bisson et al., 2010, 2012). Most studies report alterations in postural control after plantar flexor fatigue. For example, Bisson et al. (2010) found increases in sway area, amplitude and velocity during postural tasks of varying difficulty, after a fatigue protocol consisting in rising on tiptoes until exhaustion. A few studies have investigated fatigue-related changes in postural control in seniors (Moore et al., 2005; Egerton et al., 2009; Bisson et al., 2014). Bisson et al. (2014) recently showed that after plantar flexor fatigue (isometric contraction maintained until exhaustion), sway area increased more in seniors than in young adults, but only when proprioception was altered. However, most of these studies have focused on the effect of fatigue on postural control during quiet standing tasks. Little is known about the effects of muscle fatigue on dynamic postural tasks. Simoneau et al. (2006) investigated the effects of a general and moderate fatiguing exercise (fast walking on a treadmill) on dynamic postural control in young adults. Participants were asked to keep the representation of their center of pressure (COP) displayed on a computer screen (i.e., a cross) inside a box moving upward and downward. For this purpose, participants had to lean their body forward and backward in order to move the cross upward and downward respectively. The authors reported that after fatigue, participants showed an increase in the time spent outside the box and in the mean COP velocity, indicating an alteration in the performance of this balance control task. In addition, Simoneau et al. (2006) observed that muscle fatigue increased the attentional demands related to dynamic postural control (as inferred from simple reaction times, RTs), suggesting that more cognitive resources were required to maintain balance after than before fatigue.

Indeed, by potentially altering both the quality of the proprioceptive information (Forestier et al., 2002; Vuillerme and Boisgontier, 2008) and the efficiency of the motor command (Enoka and Duchateau, 2008), muscle fatigue could lead to additional attentional requirements to maintain optimal posture. Executive functions tend to deteriorate with aging, attention and working memory being the most affected functions (Glisky, 2007). These executive functions play an important role in maintaining postural balance in seniors, particularly during dual-task conditions (Tinetti et al., 1988; Woollacott and Shumway-Cook, 2002; Boisgontier et al., 2013). Using a dual-task paradigm, Lajoie et al. (1996) have reported that healthy seniors need to allocate a greater proportion of attentional resources to postural control during upright standing and walking tasks compared to young adults. Similarly, Teasdale et al. (1993) showed that in healthy seniors, altering the quality of sensory information (e.g., by adding a foam on the support surface) and/or decreasing the availability of specific sensory information (e.g., by performing the task with eyes closed) resulted in a greater increase in attentional demands associated with postural control than in younger adults. An aged-related deterioration of the peripheral sensory systems combined with poorer central integrative mechanisms could explain this increased amount of attention required to perform postural tasks (Teasdale et al., 1991, 1993). Dynamic balance tasks have shown a greater sensitivity to the age-related increase in the attentional cost of balance (Boisgontier et al., 2013). In addition, more and more balance training programs administered to seniors include dynamic postural exercise. Thus, investigating postural control in such dynamic context may be more relevant to falls.

Therefore, the purpose of this study was to investigate the effect of aging on dynamic postural control and posture-related attentional demands before and after a plantar flexor fatigue protocol. To characterize dynamic postural control, we chose a postural tracking task which consisted in following a moving target on a computer screen with the representation of the individual’s COP displayed on the screen. We hypothesized that seniors would present with a lesser ability to perform the dynamic task and would require more attention to control their COP displacements than young adults. We also expected that muscle fatigue would alter dynamic balance and increase the associated attentional demands in both young adults and seniors, with seniors potentially exhibiting a higher sensitivity to muscle fatigue (i.e., showing greater alterations in dynamic balance and higher increase in attentional demands).

## Materials and Methods

### Participants

Fifteen healthy young adults (Young group; 7 women and 8 men; 25.8 ± 4.2 years, 170.3 ± 10.3 cm and 67.6 ± 15.9 kg) and 13 healthy seniors (Senior group; 7 women and 6 men; 64.6 ± 4.2 years, 168.1 ± 10.1 cm and 74.7 ± 15.2 kg) participated in this study. None of the participants reported neurological, vestibular, or orthopedic conditions, including lower-limb injury in the 6 months prior to data collection, which could have affected postural control. No participant was under any medications known to influence balance. All participants had normal or corrected-to-normal vision. Participants were informed of the nature and aim of the study, and signed an informed consent form. The experimental protocol was approved by the Bruyère Continuing Care Research Ethics Board and the University Human Research Ethics Committee of the University of Ottawa.

### Experimental Procedure

**Figure 1:**
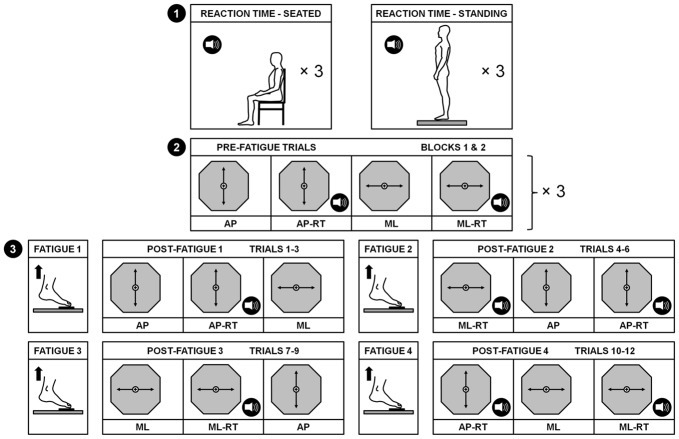
**Schematic representation of the experimental design.** Baseline reaction times (RTs) were first measured in both seated and standing positions (1). Then, 2 blocks of 12 pre-fatigue trials were randomly performed (2). These pre-fatigue trials consisted of performing a dynamic postural tracking task along the anterior-posterior (AP) and the medial-lateral (ML) axes with and without a concurrent RT task (represented by the speaker pictogram). Then, four fatiguing exercises required participants to stand up on tiptoes as long as possible with a maximal plantar flexion angle. After each fatigue exercise, 3 post-fatigue trials were performed, in a random order, until the completion of the 12 post-fatigue trials (3).

**Figure 2:**
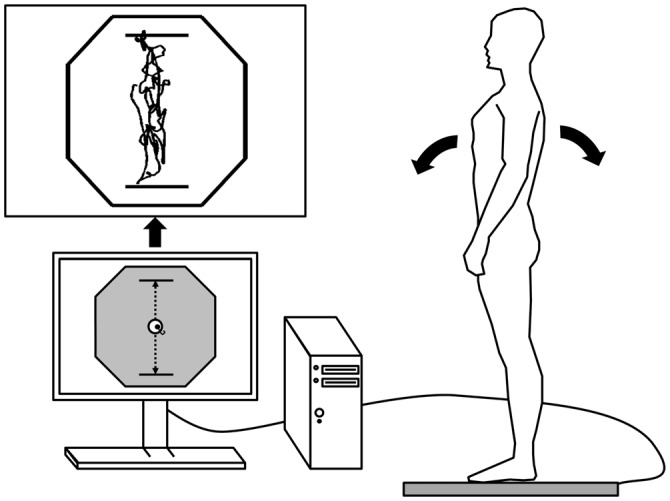
**Schematic representation of the postural tracking task during AP trials**. The center of pressure (COP) was recorded from a force platform and displayed in real-time by a cursor on a computer screen. The hexagon represented the participant’s base of support (BOS). The postural tracking task consisted in keeping the participant’s COP as close as possible from the target (i.e., a circle moving from top to bottom during AP trials or from side-to-side during ML trials) on the computer screen (adjusted at eye level). For this purpose, participants had to lean forward and backward during AP trials or from side to side during ML trials. The range of the target displacement (represented by the two dotted black arrows) was set at 80% BOS in each direction. An example of raw data obtained during an AP trial is presented on the top left corner.

A schematic representation of the protocol is shown in Figure 1. After a familiarization period with the RT measurement procedure, participants performed six baseline RT trials: three in the seated position (SEATED) and three in normal upright stance with feet shoulder width apart (STANDING). During each of these 30 s trials, six 50 ms auditory stimuli (3000 Hz) were presented at random temporal intervals in order to avoid any anticipatory effect. Participants had to provide a verbal response, the word “top”, as quickly as possible after each auditory stimulus. Then, in order to assess the participants’ limits of stability, each individual had to stand barefoot with feet together on a force platform (AccuGait System, AMTI, Watertown, MA, USA) and was instructed to lean forward and backward, and then side-to-side, as far as they could while maintaining contact between the sole of the foot and the platform at all times. Participants were instructed to move about their ankles and keep knees and hips straight. This procedure was repeated twice and care was taken to ensure that participants did not lift their feet off the platform during the task. For each direction, the maximum distance travelled by the COP was recorded via the Balance Trainer software (version 1.05, AMTI, Watertown, MA, USA) to further estimate and display a representation of the BOS on a 56 cm flat screen monitor placed ~80 cm in front of participants (Figure 2). Then, participants practiced a postural tracking task along the antero-posterior (AP) and the medio-lateral (ML) axes, twice in each direction. The postural tracking task consisted in following a target (i.e., a circle with a diameter equal to 2.5% of the BOS) moving upward and downward (four cycles) or side-to-side (seven cycles) on the screen with a cursor representing the COP of participants. The displacement range in each direction was set at 80% of the BOS, with a speed of 1.9 cm/s. The actual COP position of participants was calculated in real-time via the Balance Trainer software and displayed on the screen with a black background, the BOS being represented on the screen by an hexagon. Each dynamic postural trial started with the target centered in the hexagon. A forward displacement of the participant’s COP generated an upward movement of the cursor while a backward displacement led to a downward movement of the cursor. Left and right displacements of the COP created corresponding left and right movements of the white cursor on the screen.

The pre-fatigue protocol consisted of two identical blocks of 12 dynamic postural trials, to test for a potential learning effect. Each block comprised three trials of each of the four following conditions: (1) a simple postural tracking task condition in which participants had to move forward and backward (AP); (2) a simple postural tracking task condition in which participants had to move from left to right (ML); (3) a dual-task condition in which participants had to move forward and backward while responding “top” to auditory stimuli (AP-RT); and (4) a dual-task condition in which participants had to move from left to right while responding “top” to auditory stimuli (AP-RT). Before the start of each trial, participants were asked to position their COP in the center of the target, and were informed of which direction the target would be moving (AP or ML), and reminded of the condition (single- or dual-task). During dual-task conditions, participants were instructed to prioritize their attention on the dynamic postural task. Within-block trial order was randomized between subjects. Following the pre-fatigue protocol, participants performed a fatigue task (see below). After the fatigue task, three post-fatigue trials were performed before performing the fatigue task again, to minimize recovery and ensure a fatigue state for each post-fatigue trial. In total, participants had to perform the fatigue task four times. The post-fatigue trials followed the same order as that of the pre-fatigue trials. Overall, the testing session lasted approximately 90 min.

### Fatigue Task

For the fatigue task, participants were required to stand on the force platform. A twin axis goniometer (SG series model, Biometrics Ltd, Newport, UK) was placed on the right ankle of the participant with one end fixed with auto-adhesive tape over the distal part of the external aspect of the leg and the other end attached to the external side of the foot, along the fifth metatarsal bone. The electronic goniometer was linked to a data acquisition unit (DataLINK DLK 900, Biometrics Ltd) connected to a computer and the plantar flexion angle could be monitored online (DataLINK software version 5.0, Biometrics Ltd). Participants first had to stand on tiptoes and hold the maximal plantar flexion position for 2–3 s, so that the experimenter could read the maximal angular value reached by the participants (0° = neutral standing position with the foot on a flat surface). For each of the four fatigue exercise blocks, participants were asked to stand on tiptoes at their maximal plantar flexion angle and to maintain this position as long as they could. This task aimed to induce localized muscle fatigue of the plantar flexors. A piece of foam (thickness = 2.5 cm) was placed under both feet, at the metatarsophalangeal joint level, to reduce the discomfort due to the maintenance of this position. If necessary, participants were allowed to lightly touch an adjustable table placed besides them to maintain their balance. When the participants could no longer maintain an ankle angle ≥80% of their maximal plantar flexion amplitude, the fatigue exercise was stopped and the piece of foam was quickly removed so that post-fatigue postural trials could begin immediately. Each of the four fatigue exercise blocks (F1–F4) was timed and recorded for further analysis.

### Data Analysis

COP displacements were recorded at a sampling frequency of 50 Hz, low-pass Butterworth filtered with a 6-Hz cut-off frequency, and then analyzed using custom-made scripts developed with Matlab (The Mathworks Inc., Natick, MA, USA). The area of the BOS (in cm^2^) was calculated for each participant from the eight-points coordinate of the hexagon. The mean distance between the COP and the target center (in cm), and the mean velocity of the COP (in cm/s) were calculated for each dynamic postural trial of every participant. The mean COP-target distance was calculated by averaging the distance between the COP and the target center computed for each time point across the trial. The mean COP velocity was defined as the total distance travelled by the COP during a given trial, divided by the trial duration. Cross-correlation analyses were also performed between the COP displacements and the target displacements: COP_x_ data series were cross-correlated with the *x*-coordinates of the target displacements for ML trials and COP_y_ data series were cross-correlated with the *y*-coordinates of the target displacements for AP trials. The maximal correlation value (R_xy_) and the corresponding phase shift (τ*; Nelson-Wong et al., 2009) were calculated for every trial of each participant. A negative phase shift indicated that the COP displacement signals preceded the target center displacements (reflecting an anticipatory control mode) whereas a positive shift indicated that the COP displacement signals followed the target center displacements (reflecting a reactive control mode).

Verbal responses to the auditory stimuli were recorded using a mobile Bluetooth technology phone headset (BH-212 model; Nokia Corporation, Newmarket, ON, Canada) that was wirelessly linked to a laptop computer using Wave Pad Sound Editor (version 4.27, NCH Software Pty Ltd, Canberra, Australia). RT performance corresponded to the verbal response latency (i.e., start of the verbal response—start of stimulus; in ms) and was computed with Matlab using a semi-automatic method (Remaud et al., 2013). Briefly, the start of the stimulus and the start of the verbal response were detected when baseline audio signal exceeded a threshold of mean baseline audio signal ± 5 standard deviations (*SD*s). RT deemed too slow to represent a true RT, i.e., those >750 ms, were first discarded from the analysis (*n* = 72). Then, Grubb’s test was used to detect potential outliers in RT data (*n* = 77). Overall, 3.5% of RT data recorded in dual-task condition were discarded from the analysis.

### Statistical Analysis

The area of the BOS and maximal plantar flexion amplitude were compared between young adults and seniors using unpaired *t*-test to investigate potential age-related changes in postural stability and range of motion. The Levenne’s test was performed to assess the homogeneity of variance assumption between the two groups. A two-way mixed design analysis of variance (ANOVA; group [senior; young] × time [F1; F2; F3; F4]) was performed on fatigue task duration to analyze potential age-related differences in time to exhaustion. Since preliminary analyses showed a learning effect on mean COP-target distance between the two blocks of pre-fatigue trials, only data from the second block were considered as the pre-fatigue condition for the subsequent analyses pertaining to mean COP-target distance, mean COP velocity and RT. Postural trials performed along the AP and ML axes were analyzed separately. Three-way mixed design ANOVAs (group × time [PRE; POST] × task condition [simple; dual]) were performed on mean COP velocity and mean COP-target distance in order to investigate potential age-related changes in dynamic postural control according to the fatigue status and the level of attentional load. Three-way mixed design ANOVAs (group × time × task condition) were also performed on R_xy_ and τ* to respectively investigate potential age-related changes in (1) the maximal degree of association between participants’ COP displacement and target displacement, and (2) the corresponding phase shift (indicating anticipatory or reactive postural control), according to the fatigue status and the level of attentional load. To confirm that the attentional load associated with posture increased from a seated posture to a dynamic standing posture, a two-way ANOVA (group × posture [seated; static standing; dynamic standing]) was performed on RT. A two-way ANOVA (group × time) was then conducted on RT collected during dynamic AP and ML trials in order to analyse potential age-related changes in attentional demands associated with dynamic postural control according to the fatigue status. When the sphericity assumption in repeated measures ANOVAs was violated (Mauchly’s test), a Geisser/Greenhouse correction was used. If relevant, *post hoc* tests were performed by means of the Newman-Keuls procedures. Statistical significance was set at *p* < 0.05. All results presented below are mean ± *SD*. Partial eta squared values (${\text{η}}_{p}^{2}$) were reported to indicate small (≥0.01), medium (≥0.06), and large (≥0.14) effect sizes (Sink and Stroh, 2006).

## Results

### Aging Effect on Base of Support

Overall, seniors showed a significantly reduced (−21%) BOS area compared to young adults (158.8 ± 46.6 cm^2^ vs. 201.9 ± 45.6 cm^2^; *t*_(26)_ = 2.47, *p* = 0.020; Levenne’s test: *F*_(1,26)_ = 0.33, *p* = 0.572).

### Aging Effect on Muscle Fatigue

First, we observed that young participants tended to have a greater maximal plantar flexion amplitude than seniors. However, this difference did not reach statistical significance (45.9 ± 10.5° vs. 39.4 ± 8.0°, respectively; *t*_(26)_ = 1.81, *p* = 0.082; Levenne’s test: *F*_(1,26)_ = 1.42, *p* = 0.244). Regarding fatigue duration, results did not show any group effect (*F*_(1,26)_ = 2.60; *p* = 0.119; ${\text{η}}_{p}^{2}$ = 0.09), but a significant time effect (*F*_(3,78)_ = 7.74; *p* = 0.008; ${\text{η}}_{p}^{2}$ = 0.23) was found. On average, the duration of the first fatiguing exercise block (347 ± 368 s) was longer than the three subsequent fatiguing exercise blocks (169 ± 91 s, 166 ± 87 s and 167 ± 91 s, respectively; *p* < 0.001), which were not different from each other. No group × time interaction was found (*F*_(3,78)_ = 1.84; *p* = 0.147; ${\text{η}}_{p}^{2}$ = 0.07).

### Aging Effect on Dynamic Postural Control

#### Mean Distance Between the COP and the Target Center

**Figure 3:**
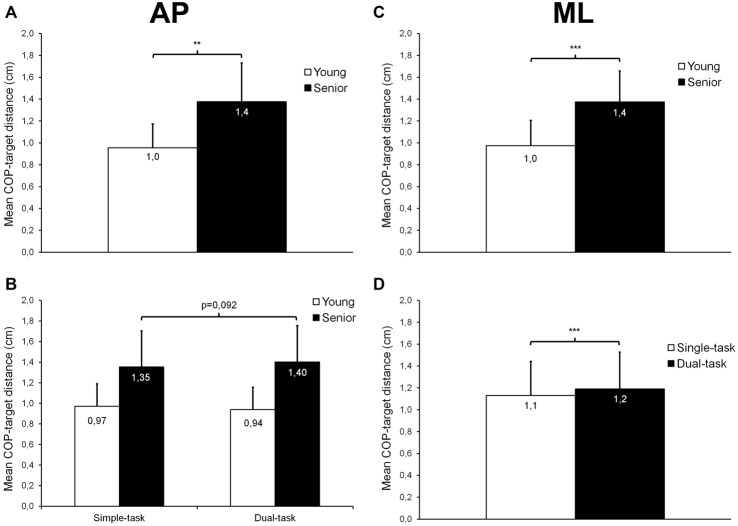
**Mean (±*SD*) COP-target distance observed during AP and ML trials**. A significant age effect was found for trials performed along the AP **(A)** and ML **(C)** axes, with seniors showing, overall, a larger COP-target distance than young adults. For AP trials, a trend toward a group × task interaction was also noticed **(B)**. For ML trials **(D)**, a slight but significant task effect was found, with dual-task condition trials showing, overall, a greater COP-target distance than single-task trials. **,***Significantly different at *p* < 0.01 and *p* < 0.001, respectively.

For AP trials, results revealed a main group effect (*F*_(1,26)_ = 16.16; *p* < 0.001; ${\text{η}}_{p}^{2}$ = 0.38; Figure 3A) on mean COP-target distance, indicating that the mean distance between the COP and the target center was 44% larger in seniors than in young participants (*p* = 0.001). No time effect was observed (*p* = 0.467). Although not statistically significant, we also noticed a trend toward a group × task interaction (*F*_(1,26)_ = 4.21; *p* = 0.0503; ${\text{η}}_{p}^{2}$ = 0.14; Figure 3B). The mean distance between the COP and the target center tended to be larger in dual-task compared with single-task in seniors (*p* = 0.092), whereas the distance remained similar in both conditions in young adults (*p* = 0.261).

For ML trials, we found main effects of group (*F*_(1,26)_ = 18.95; *p* < 0.001; ${\text{η}}_{p}^{2}$ = 0.42; Figure 3C) and task (*F*_(1,26)_ = 18.84; *p* < 0.001; ${\text{η}}_{p}^{2}$ = 0.42; Figure 3D) on mean COP-target distance. The mean distance between the COP and the target center was 41% larger in seniors than in young participants (*p* < 0.001), and was also 5% greater during dual- than single-task condition (*p* < 0.001). No time effect was noted (*p* = 0.507).

#### Mean Velocity of the COP Displacements

**Figure 4:**
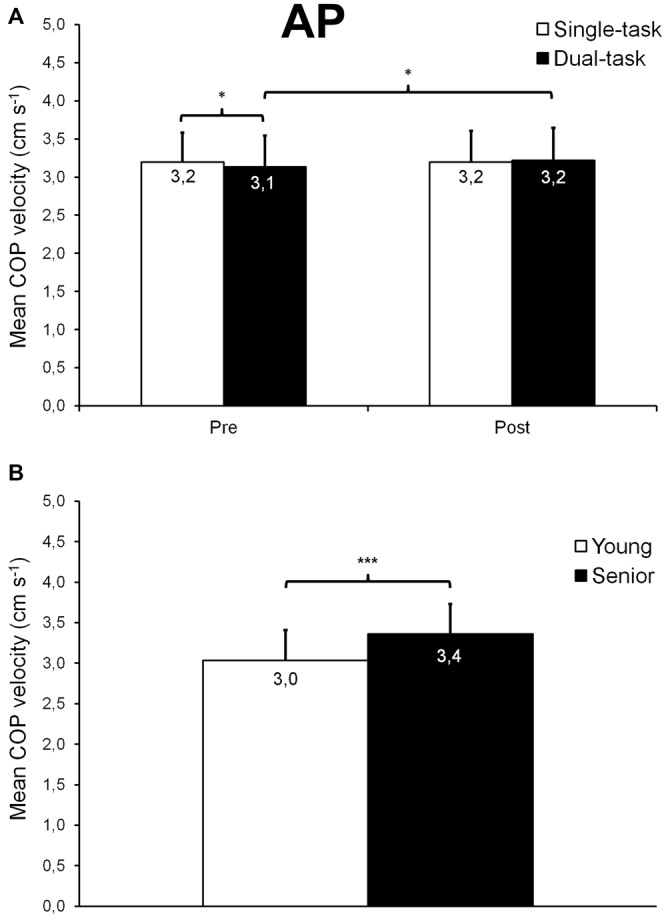
**Mean (±*SD*) COP velocity observed during AP trials**. A significant time × task interaction was found **(A)**, showing a slightly slower COP velocity in dual- compared with single-task condition trials before fatigue, as well as a small COP velocity increase after fatigue for dual-task condition trials. A significant age effect was also noticed **(B)**, seniors exhibiting, overall, a faster COP velocity than young adults. *,***Significantly different at *p* < 0.05 and *p* < 0.001, respectively.

For AP trials, we found a time × task interaction (*F*_(1,26)_ = 4.24; *p* = 0.0497; ${\text{η}}_{p}^{2}$ = 0.14; Figure 4A) on mean COP velocity, as well as a main group effect (*F*_(1,26)_ = 6.10; *p* = 0.020; ${\text{η}}_{p}^{2}$ = 0.19; Figure 4B). In dual-task condition, mean COP velocity was 3% higher after than before fatigue (*p* = 0.027) whereas it remained unchanged in single-task condition (*p* = 0.972). However, mean COP velocity was 2% slower in dual- than single-task condition before fatigue (*p* = 0.039) while after fatigue, mean COP velocity was similar for both conditions (*p* = 0.428). Also, overall, seniors exhibited a 11% higher mean velocity of the COP displacements than young participants (*p* = 0.021).

For ML trials, our statistical analysis revealed no main effect of group (*p* = 0.760), time (*p* = 0.239) or task (*p* = 0.671) and no interaction between these factors.

#### Cross-Correlation Analyses

**Figure 5:**
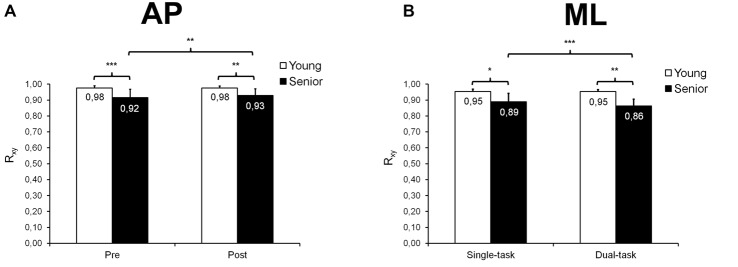
**Mean (±*SD*) maximal correlation coefficient (R_xy_) derived from cross-correlation analyses and observed during AP and ML trials**. A significant group × time interaction was found for AP trials **(A)**, seniors exhibiting, overall, lower R_xy_ values than young adults before and after fatigue, and showing a small increase in R_xy_ after than before fatigue. A significant age × task interaction was also noticed for ML trials **(B)**, R_xy_ being, overall, lower in seniors than in young adults before and after fatigue, and seniors showing smaller R_xy_ values in dual- than single-task condition. *,**,***Significantly different at *p* < 0.05, *p* < 0.01 and *p* < 0.001, respectively.

**Figure 6:**
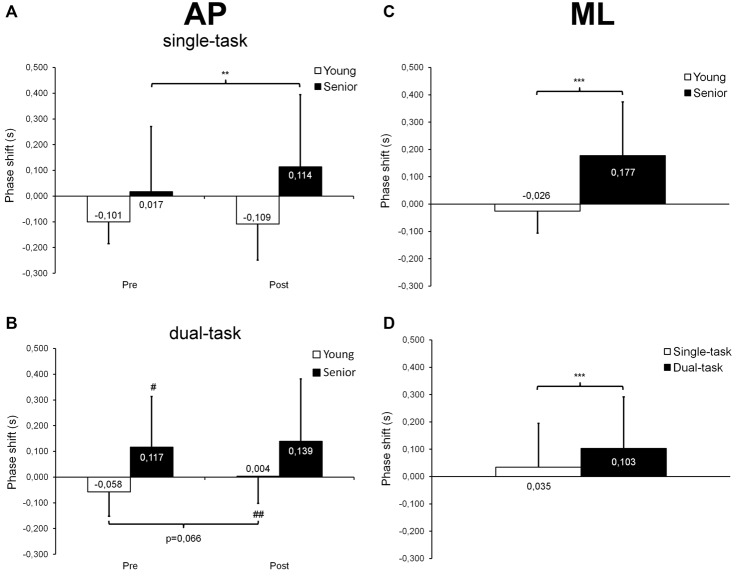
Mean (±*SD*) phase shift (τ*) derived from cross-correlation analyses and observed at maximal correlation coefficient during AP and ML trials. A negative phase shift indicates that the COP displacement signals preceded the target center displacements (anticipatory control mode) whereas a positive shift indicates that the COP displacement signals followed the target center displacements (reactive control mode). For AP trials, a significant group × time × task interaction was found **(A,B)**. In single-task condition, seniors showed a longer positive shift after than before fatigue **(A)** whereas in dual-task condition, the negative shift observed in young adults tended to become slightly positive with fatigue **(B)**. Also, seniors presented with a longer positive shift in dual- than single-task condition before fatigue. After fatigue, young adults switched from a negative shift in single-task condition to a slightly positive shift in dual-task condition. For ML trials, a significant age effect was noticed **(C)**, seniors showing a positive shift while young adults exhibiting a negative shift. A significant task effect was also observed **(D)**, with longer positive shift being observed in dual- than single-task condition. ^#,^^##^Significantly different from the single-task condition at *p* < 0.05 and *p* < 0.01, respectively. **,***Significantly different at *p* < 0.01 and *p* < 0.001, respectively.

Regarding AP trials, our statistical analysis revealed a significant group × time interaction (*F*_(1,26)_ = 4.69; *p* = 0.040; ${\text{η}}_{p}^{2}$ = 0.15; Figure 5A) on R_xy_, as well as main effects of group (*F*_(1,26)_ = 20.75; *p* < 0.001; ${\text{η}}_{p}^{2}$ = 0.44) and time (*F*_(1,26)_ = 5.27; *p* = 0.030; ${\text{η}}_{p}^{2}$ = 0.17). Seniors showed lower R_xy_ values than young adults before (*p* < 0.001) and after (*p* = 0.002) fatigue. Surprisingly, seniors demonstrated higher R_xy_ values after than before fatigue (*p* = 0.004) whereas young adults showed similar R_xy_ values before and after fatigue (*p* = 0.928). The ANOVA on τ* revealed a group × time × task interaction (*F*_(1,26)_ = 5.08; *p* = 0.033; ${\text{η}}_{p}^{2}$ = 0.16; Figures 6A,B), as well as main effects of group (*F*_(1,26)_ = 6.81; *p* = 0.015; ${\text{η}}_{p}^{2}$ = 0.21), time (*F*_(1,26)_ = 5.22; *p* = 0.031; ${\text{η}}_{p}^{2}$ = 0.17) and task (*F*_(1,26)_ = 13.02; *p* = 0.001; ${\text{η}}_{p}^{2}$ = 0.33). In the single-task condition, young adults showed a similar negative phase shift (i.e., an anticipatory control mode) before and after fatigue (*p* = 0.796) whereas seniors demonstrated a longer positive shift (i.e., a reactive control mode) after than before fatigue (*p* = 0.006; Figure 6A). In dual-task condition, young adults tended to shift from an anticipatory control mode before fatigue to a slightly reactive control mode after fatigue (*p* = 0.066), whereas no significant difference was observed before and after fatigue in seniors (*p* = 0.486; Figure 6B). During post-fatigue trials, young participants exhibited a significant change in τ*, switching from an anticipatory control mode in single-task condition to a slightly reactive control mode during dual-task condition (*p* = 0.008). In contrast, during pre-fatigue trials, seniors showed a longer positive shift in dual- than single-task condition (*p* = 0.012). Although no significant difference was observed between young and senior participants when each single condition was considered (pre-fatigue/single-task, pre-fatigue/dual-task, post-fatigue/single-task, post-fatigue/dual-task), we did found a main group effect indicating that, overall, seniors used a significantly different control mode than young participants (τ*= 0.097 ± 0.242 s vs. −0.066 ± 0.115 s; *p* = 0.015).

Regarding ML trials, our statistical analysis indicated a significant group × task interaction (*F*_(1,26)_ = 10.04; *p* = 0.004; ${\text{η}}_{p}^{2}$ = 0.28; Figure 5B) on R_xy_, as well as main effects of group (*F*_(1,26)_ = 13.43; *p* = 0.001; ${\text{η}}_{p}^{2}$ = 0.34), time (*F*_(1,26)_ = 4.45; *p* = 0.045; ${\text{η}}_{p}^{2}$ = 0.15) and task (*F*_(1,26)_ = 11.46; *p* = 0.002; ${\text{η}}_{p}^{2}$ = 0.31). Seniors showed lower R_xy_ values than young adults in both single-task (*p* = 0.014) and dual-task (*p* = 0.001) conditions. In addition, seniors demonstrated decreased R_xy_ values in dual-task compared with single-task condition (*p* < 0.001) whereas young adults exhibited similar R_xy_ values in both conditions (*p* = 0.880). Furthermore, our results revealed main effects of group (*F*_(1,26)_ = 19.82; *p* < 0.001; ${\text{η}}_{p}^{2}$ = 0.43; Figure 6C) and task (*F*_(1,26)_ = 26.47; *p* < 0.001; ${\text{η}}_{p}^{2}$ = 0.50; Figure 6D) on τ*. Seniors used a reactive control mode whereas young participants used a rather anticipatory control mode (*p* < 0.001). Moreover, the phase shift between COP and target center displacements was generally higher in dual-task than in single-task conditions (*p* < 0.001).

### Aging Effect on Reaction Time During Dual-Task

**Figure 7:**
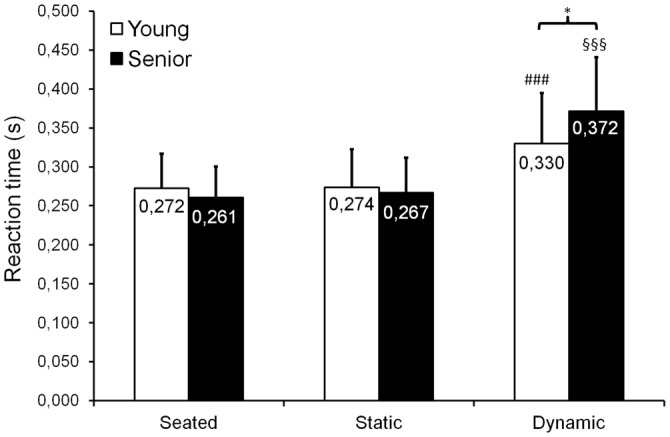
**Mean (±*SD*) reaction time (RT) observed during seated, static standing and dynamic standing (AP and ML trials are pooled) conditions**. A significant group × posture interaction was found, showing an increased RT in the dynamic standing condition compared with the seated and static standing conditions for both young adults and seniors. Moreover, overall, seniors presented with longer RTs than young adults in the dynamic standing condition. ^###,^^§§§^Significantly different from the seated and static standing conditions, respectively for young adults and seniors, at *p* < 0.001. *Significantly different at *p* < 0.05.

When comparing seated, static standing and dynamic standing conditions, we observed a significant group × posture interaction for RT (*F*_(2,52)_ = 8.98; *p* < 0.001; ${\text{η}}_{p}^{2}$ = 0.26; Figure 7), as well as a main posture effect (*F*_(2,52)_ = 93.84; *p* = 0.004; ${\text{η}}_{p}^{2}$ = 0.78). Mean RT remained similar between seated and static standing conditions for both young adults (*p* = 0.898) and seniors (*p* = 0.562). No significant difference in mean RT was observed between young adults and seniors, for both the seated (*p* = 0.831) and the static standing (*p* = 0.931) conditions. During dynamic standing condition, mean reaction time significantly increased when compared to seated and static standing for both young adults (*p* < 0.001) and seniors (*p* < 0.001). We also found that during dynamic standing, mean reaction time was longer for seniors than for young adults (*p* = 0.047).

When now focusing only on dynamic standing conditions, no significant main effect or interaction between group and time factors (*p* > 0.05) was observed for both AP and ML trials.

## Discussion

In the present study, we aimed to compare the effects of a plantar flexor fatigue protocol on dynamic postural control and posture-related attentional demands between young adults and seniors. For this purpose, we used a postural tracking task consisting in following a moving target on a computer screen with a cursor representing the COP by leaning forward and backward or side-to-side. In half of the trials, a secondary auditory-verbal RT task was added to probe the attentional demands associated with dynamic postural control. Overall, our findings indicate that seniors demonstrated poorer dynamic postural control than young adults. Muscle fatigue and the addition of a secondary task slightly altered dynamic postural control in both young adults and seniors. Interestingly, parameters derived from cross-correlation analyses between COP displacements and target displacements appeared more sensitive to aging, fatigue and dual-tasking than traditional sway parameters, thus providing useful information about the postural strategy used by each age group (see below). In addition, performing the dynamic standing task significantly increased the attentional demands associated with postural control in both age groups, but this increase was more pronounced in seniors.

### Aging Effect on Dynamic Postural Control

We first observed that the BOS area was 21% smaller in seniors than in young adults. Complementary correlation analyses showed that BOS area was not correlated with weight (*p* > 0.05), was close to be significantly correlated with height (*r* = 0.37; *p* = 0.050), and was negatively correlated with age (*r* = −0.44; *p* = 0.021). This confirms the results previously obtained by Duncan et al. (1990) and King et al. (1994) who reported that both age and height were the most significant factors influencing the AP limits of stability. In the present study, we extended this result to the whole BOS area. Since height was not statistically different between our two groups, it is likely that the BOS area difference observed in the present study was mainly due to the aging process. Age-related decline in proprioception (Goble et al., 2009), which may prevent seniors from accurately detecting their limits of stability, as well as a decreased ability to generate rapid ankle joint torques to recover balance (Thelen et al., 1996), could partly explain this result.

During the postural tracking task, young and senior participants were asked to follow the target with the representation of their COP on the computer screen to the best of their ability. To assess their performance, we calculated the mean COP-target distance and found that, overall, seniors performed worse than young participants in both AP (+44%) and ML (+41%) directions (see Figures 3A,C). At the same time, during AP trials, seniors showed an increased mean COP velocity (+11%) compared with young adults (see Figure 4B), suggesting a greater need for corrective actions in the former group. In addition, the cross-correlation analyses showed that, for both AP and ML trials, the COP displacements were less correlated with the target displacements in seniors than in young adults (see Figures 5A,B). All these results demonstrate that the ability to control balance during dynamic standing conditions significantly decreases with aging. Other studies have reported age-related alterations in the control of balance during dynamic standing conditions (Bugnariu and Sveistrup, 2006; Karamanidis et al., 2008; Mademli et al., 2008), although the experimental paradigms of these studies were somewhat different. For example, Mademli et al. (2008) found that when recovering balance from a forward fall, seniors stepped with a narrower anterior boundary of their BOS than young adults, this resulting in a lower margin of stability. The authors explained this finding by a lower ability of seniors to rapidly increase their BOS due to a lower rate of force generation and/or a weaker hip flexion moment during the initial phase of balance recovery. Bugnariu and Sveistrup (2006) compared the postural strategies used by young adults and seniors to cope with sinusoidal AP postural perturbations induced by a moving platform. They noticed that when the frequency of oscillations was high (i.e., 0.5–0.61 Hz), the COP range observed in seniors was larger and located more often in less safe regions (i.e., close from the boundaries of the BOS) than young adults. Seniors also had to take more steps than young adults to recover balance. These age-related alterations in the control of dynamic balance could be explained by neuromuscular factors such as a decrease in plantar cutaneous sensitivity (Meyer et al., 2004; Araneda and Solorza, 2013) and/or a lower capacity to generate and control muscle force, especially at the ankle level (Melzer et al., 2009).

In our study, cross-correlation analyses also revealed that the maximal correlation between COP and target displacement time-series was obtained with a positive time delay in seniors, indicating a reactive control mode, whereas this time delay was negative in young adults, suggesting an anticipatory control mode (see Figures 6A–C). In their study, Bugnariu and Sveistrup (2006) investigated cross-correlation time delays between the platform movements (i.e., sinusoidal AP perturbations) and the COP displacements of young and senior participants. Participants had to adapt to changes in the frequency of the platform movements, which could be either self- triggered or externally-triggered. Their results showed that seniors presented with longer time delays than young adults when the frequency change was externally-triggered. However, both age groups exhibited a reactive control mode (i.e., the COP displacements always lagged behind the platform movements), which was expected given their experimental paradigm. In the present study, the target movement was predictable. Indeed, participants had to synchronize their COP displacements with those of a target moving upward and downward or side-to-side on a screen at a regular speed and following a constant trajectory. Therefore, participants generated self-induced postural perturbations and time delays derived from cross-correlations could thus be either slightly negative (i.e., the COP displacements preceded the target movement) or slightly positive (i.e., the COP displacements lagged behind the target movement), depending on their ability to control their COP and to anticipate the target movement. Although the age-related difference observed in this study could be related to alterations in proprioception and/or lower-leg muscle strength (as mentioned above), a decreased capacity to process sensory information (Boisgontier and Nougier, 2013) and/or to allocate appropriate attentional resources to the dynamic balance task may also explain this result.

Our findings revealed that overall (i.e., when young and senior participants are pooled), mean RT increased when switching from a seated or static standing task to a dynamic standing task (see Figure 7). This indicates that performing a dynamic balance task requires more attentional demands than remaining seated or than standing quietly. However, we observed that this increase in mean RT was more pronounced in seniors than in young adults, suggesting that seniors required more attention to perform the dynamic balance task than young adults. Our results confirm those previously obtained in the literature (for a review, see Boisgontier et al., 2013), who showed that in seniors, increasing postural task difficulty resulted in a higher increase in attentional demands compared with young adults. Poorer central integrative mechanisms in seniors have been hypothesized to explain this result (Teasdale et al., 1991, 1993). Alternatively, the longer RTs observed in seniors during dynamic balance tasks could also be explained by an age-related slowing of the CNS functions, as well as by disruptions in decision-making process and in higher cortical functions. Indeed, Fozard et al. (1994) reported that from the age of 20, aging implies a continuous slowing of RT, which is emphasized when task complexity is increased.

### Fatigue Effect on Dynamic Postural Control

First, our results showed that no difference in fatigue duration occurred between young adults and seniors. Previous studies have found greater fatigue resistance in seniors compared with young adults, when the fatigue task was performed either in isometric or isokinetic condition (Bilodeau et al., 2001; Lanza et al., 2004; Hunter et al., 2005). To explain this result, Kent-Braun (2009) suggested that the combination of lower motor unit discharge rate, slower contractile properties and relatively greater reliance on oxidative metabolism observed in seniors during fatiguing exercise would confer them a greater resistance to muscle fatigue than young adults. However, this difference in fatigue resistance between young adults and seniors is not always observed (Lindström et al., 1997; Allman and Rice, 2001), which may suggest that this difference is task-dependant (Enoka and Stuart, 1992). In the present study, participants had to stand up on tiptoes with a maximal plantar flexion angle until exhaustion, which has been previously reported to alter postural control in quiet stance (Vuillerme et al., 2001, 2002). Since the muscle torque developed at the ankle level mainly depended on the weight of the participant and with a similar mean weight observed in both age groups, the ankle torque likely represented a same relative force level for both groups.

Moreover, we observed that overall, fatigue of the plantar flexor muscles only had a minor effect on dynamic postural control. We noticed a small increase (+3%) in mean COP velocity after fatigue, but only during dual-task trials and without any significant difference between young adults and seniors (see Figure 4B). In addition, during single-task AP trials, seniors exhibited a longer phase shift (i.e., a slower reactive control mode) after than before fatigue (see Figure 6A). However, no changes were observed for mean COP-target distance and RT, this indicating that the overall performance of the dynamic balance task and the posture-related attentional demands were not altered by plantar flexor muscle fatigue. Numerous studies have reported alterations of static postural control in young adults after plantar flexor fatiguing exercises (Vuillerme et al., 2002; Yaggie and McGregor, 2002; Gribble and Hertel, 2004), including previous works from our laboratory (Boyas et al., 2011, 2013; Bisson et al., 2012). In seniors, this effect of local muscle fatigue on static postural control appears to be more pronounced than in young adults (Bisson et al., 2014). However, when considering studies that investigated the effects of muscle fatigue on dynamic postural control, results appeared mitigated, even in seniors (Simoneau et al., 2006; Mademli et al., 2008; Egerton et al., 2010; Kennedy et al., 2012; Toebes et al., 2014). For example, Simoneau et al. (2006) reported a decrease in dynamic postural control of young adults after a first moderate fatiguing exercise (fast walking on a treadmill). But they noted that their participants were able to rapidly compensate for the effect of fatigue after the two subsequent fatigue periods, by increasing the frequency of actions. In contrast, Egerton et al. (2010) tested dynamic postural stability of young adults and healthy seniors, as well as balance-impaired seniors before and after a 14 min bout of moderate-intensity physical exercise. They reported an improved coordination of the step task after exercise, showing that postural stability was not deteriorated immediately after fatigue, even among balance-impaired seniors. Recently, Toebes et al. (2014) analyzed balance control in healthy seniors during perturbed and unperturbed gait before and after unilateral muscle fatigue of the knee extensors. They observed no balance impairment after fatigue, regardless of the gait condition. Moreover, they found that when confronted with laterally directed mechanical perturbations, healthy seniors recovered their balance faster after fatigue. It is likely that the duration of the fatigue protocol and the muscle groups involved in the fatigue task play an important role in the potential changes observed in dynamic postural control after fatigue. In the present study, our fatigue protocol targeted only the plantar flexor muscles for relatively short periods of time (i.e., 3–6 min on average), although a high-intensity level of muscle contraction was expected. This resulted in marginal alterations in dynamic postural control, although seniors tended to adopt a slower reactive control mode. Future studies should aim to clarify why the fatigue-related impairments in postural control observed during quiet stance does not transfer to the dynamic conditions. For this purpose, researchers may compare the effects of a given fatigue protocol on both static and dynamic postural tasks.

### Dual-Tasking Effect on Dynamic Postural Control

The results of the present study showed that during ML trials, participants’ COP was, on average, 5% further from the target center in dual-task condition than in single-task condition (see Figure 3D). During AP trials, only seniors tended to show a greater COP-target distance (+4%; *p* = 0.092) in dual- compared with single-task conditions (see Figure 3B). These results indicate that dual-tasking increased the difficulty of the dynamic postural task, particularly when trials were performed in the ML direction, but in a minor way. Also, before fatigue, mean COP velocity was 2% slower in dual- than single-task condition but this difference was not observed after fatigue (see Figure 4A). Given the magnitude of this decrease and the fact that it only occurred before fatigue, it is not possible to confirm a dual-task effect on mean COP velocity.

Furthermore, cross-correlation analyses showed that in seniors, the maximal correlation value observed between the COP displacements and the target movement during ML trials was lower in dual-task (R_xy_ = 0.86) than in single-task (R_xy_ = 0.89) condition (see Figure 5B), suggesting a detrimental effect of dual-tasking on seniors’ performance. Concurrently, seniors showed a 68 ms increase in phase shift between dual- and single-task ML trials (see Figure 6D). During AP trials, seniors exhibited a 100 ms longer positive shift in dual- than single-task condition, but only before fatigue (see Figures 6A,B). No difference was found between dual- and single-task conditions after fatigue. This absence of difference may have resulted from muscle fatigue that already resulted in a 97 ms increase in the phase shift during single-task trials. In contrast, young participants switched from a negative phase shift (−58 ms) in single-task to a slightly positive phase shift (+4 ms) in dual-task during post-fatigue AP trials, indicating a less anticipatory dynamic postural control. As no difference was observed during pre-fatigue trials, this finding suggests that in young adults, the combination of both muscle fatigue and dual-tasking is necessary to alter dynamic postural control.

In conclusion, this study showed that compared with young adults, seniors demonstrated alteration in dynamic postural control during the postural tracking task. While young adults adopted an anticipatory control mode to move their COP as close as possible to the center of the moving target, seniors adopted a reactive control mode, following the target center with lag. This resulted in a longer mean COP-target distance, a higher COP velocity and a weaker correlation between COP displacements and target displacements in seniors, which overall indicated a poorer performance. Concurrently, the attentional demands associated with postural control increased more from seated or static standing to dynamic standing condition in seniors than in young adults, suggesting potential alterations in CNS functions. Finally, it appears that contrary to our hypothesis, fatigue of the plantar flexor muscles and dual-tasking have only minor effects on dynamic postural control of both young adults and seniors. Future studies should investigate why the fatigue-related alterations in postural control observed during quiet standing tasks do not seem to transfer to dynamic balance tasks. This would allow a better understanding of the potential risks of muscle fatigue and/or dual-tasking on balance control that are carried by seniors in daily activities.

## Author Contributions

AR conceived and designed the study, carried out data collection, analyzed and interpreted the data, and drafted the manuscript. CT-C designed the study, carried out data collection, assisted with data analysis and revised the manuscript. MB conceived and designed the study, interpreted the data and revised the manuscript. All the authors read and approved the final manuscript.

## Funding

This study was funded in part by the Natural Sciences and Engineering Research Council of Canada (Discovery Grant # 312041-2008 to M. Bilodeau) and the Canada Foundation for Innovation (Leaders Opportunity Fund Award to M. Bilodeau).

## Conflict of Interest Statement

The authors declare that the research was conducted in the absence of any commercial or financial relationships that could be construed as a potential conflict of interest.
